# Study of the Mechanical Environment of Chondrocytes in Articular Cartilage Defects Repaired Area under Cyclic Compressive Loading

**DOI:** 10.1155/2017/1308945

**Published:** 2017-07-09

**Authors:** Hai-Ying Liu, Hang-Tian Duan, Chun-Qiu Zhang, Wei Wang

**Affiliations:** ^1^Tianjin Key Laboratory of the Design and Intelligent Control of the Advanced Mechatronical System, Tianjin University of Technology, Tianjin 300384, China; ^2^Department of Mechanics, School of Mechanical Engineering, Tianjin University, Tianjin 300354, China

## Abstract

COMSOL finite element software was used to establish a solid-liquid coupling biphasic model of articular cartilage and a microscopic model of chondrocytes, using modeling to take into account the shape and number of chondrocytes in cartilage lacuna in each layer. The effects of cyclic loading at different frequencies on the micromechanical environment of chondrocytes in different regions of the cartilage were studied. The results showed that low frequency loading can cause stress concentration of superficial chondrocytes. Moreover, along with increased frequency, the maximum value of stress response curve of chondrocytes decreased, while the minimum value increased. When the frequency was greater than 0.2 Hz, the extreme value stress of response curve tended to be constant. Cyclic loading had a large influence on the distribution of liquid pressure in chondrocytes in the middle and deep layers. The concentration of fluid pressure changed alternately from intracellular to peripheral in the middle layer. Both the range of liquid pressure in the upper chondrocytes and the maximum value of liquid pressure in the lower chondrocytes in the same lacunae varied greatly in the deep layer. At the same loading frequency, the elastic modulus of artificial cartilage had little effect on the mechanical environment of chondrocytes.

## 1. Introduction

Articular cartilage is a layer of load-bearing hydrated soft tissue with low friction, covering the joint surfaces of both ends of long bones. Its mechanical properties and morphology are maintained by the metabolism of chondrocytes [1, 2]. Normal articular cartilage can bear many millions of cycles of high force loading [3], but with exercise of increasing energy and force, the problem of aging becomes increasingly serious. Approximately 65% of the total population had suffered injury to their articular cartilage [4]. Because of the lack of blood vessels, nerves, and lymphatic tissue, cartilage cannot easily repair itself after injury. In recent years, tissue engineering techniques to repair articular cartilage defects have attracted much attention [4, 5]. This technology is expected to be the most effective method to cure the cartilage defect. Until now, using tissue engineering techniques for repairing cartilage defects result in a clinical therapeutic success rate of approximately 50%, having a good short-term effect but uncertain long-term results due to the occurrence of the graft degeneration, degenerescence, joint cracking, hardening, or even the induction of the degeneration of the host cartilage [2, 6, 7]. The main reason is that the mechanical properties of tissue-engineered cartilage are not comparable to those of natural cartilage, and implants will alter the mechanical environment of the solid matrix and chondrocytes within the zone of the repair. Chondrocytes respond to stress stimuli by changing shape. Deviations from physiological load affect the gene expression, metabolism, and also the possible induction of apoptosis of chondrocytes [2, 8]. Experimental results of single chondrocytes in vitro indicate that cyclic loading affects chondrocyte biosynthesis more significantly than that of static loading [9]. Studies have shown that the specific frequency of cyclic loading can increase the aggrecan synthesis in cartilage explants [10]. Thus, following clinical tissue-engineered cartilage repair, patients are advised to gradually exercise during hospitalization and after discharge [2]. Appropriate rehabilitation exercises may alleviate pain and improve the metabolism of articular chondrocytes.

At present, the principal method used to study the mechanical properties of chondrocytes is to perform in vitro experiments or finite element numerical simulation [11–14] and to directly measure parameters such as cell deformation, elastic modulus, or surface potential. However, the results do not really reflect the micromechanical environment of chondrocytes in vivo. Until now, it has been difficult to monitor the cell volume, stress, and strain or changes in the pressure of intracellular fluid in chondrocytes during cyclic loading [15]. A multiscale study of the biomechanical environment of chondrocytes by finite element analysis could satisfy the requirements for evaluating these measurements. Zhou et al. used multiscale methods to study the distribution of stress and the flow field of fluid in cartilage under static loading and the role of pericellular matrix (PCM) in protecting chondrocytes from high stress damage [16]. Tauska et al. also used multiscale methods to study the mechanical behavior of articular chondrocytes during normal and medial meniscectomy [17]. Guilak and Haider established a multiscale linear biphasic cell-matrix interaction model of articular cartilage, with which the complex correlation between the dynamic environment of chondrocytes and their macroscopic load characteristics under confined compression cyclic load was studied [8]. Erdemir et al. used multiscale methods to establish a high-throughput model of joints, with information transfer being established between different scales generating a simulation study of changes in the mechanical environment in cell scale under macro loading [18].

The mechanical response of chondrocytes to cyclic compression depends on the amplitude and frequency of the load [8, 15, 19]. In this paper, COMSOL finite element software was used to establish an unconfined model of articular cartilage with full-thickness defects and a transscale chondrocyte model. The influence of frequency in cyclic compressive loading and the elastic modulus of artificial cartilage on the micromechanical environment of the chondrocytes in a repaired defect was studied by numerical simulation.

## 2. Materials and Methods

COMSOL Multiphysics (3.5a; Royal Institute of Technology, Stockholm, Sweden), a finite element software, was selected to simulate the mechanical environment of chondrocytes in three steps. The first step established the finite element model of cartilage and chondrocytes. The second step calculated the displacement and stress fields of articular cartilage, so as to provide accurate boundary conditions for the chondrocyte model. The third step mapped the results of the corresponding region of the cartilage model onto the chondrocyte model using consistent boundary conditions, to accomplish data conversion.

### 2.1. Articular Cartilage Model

In the 1980s, Mow et al. established a solid-liquid coupling biphasic porous medium cartilage model [20]. The model accurately reflects the macroscopic mechanical properties of cartilage because the characteristics of fiber reinforced were considered.

#### 2.1.1. Material Parameters

The material parameters of cartilage anisotropy, Poisson's ratio *ν* and the elastic modulus *E*, are related in a depth-dependent manner as follows [21–23]:

(1) $$\begin{array}{c} \nu =0.08+0.1\left(\frac{h-y}{h}\right), \end{array}$$

(2) $$\begin{array}{c} E\left(y\right)=\frac{3.66}{46.2e^{-6.53y}+2.84}, \end{array}$$

where *h* and *y* are thickness and depth of cartilage, respectively.

Cartilage permeability *k* is a nonlinear function related to depth and deformation [23, 24]:

(3) $$\begin{array}{c} k=k_{0}\psi \left(y,h\right)\exp\left(M\varepsilon_{v}\right), \end{array}$$

where *k*_0_ = 2 × 10^−15^m^4^/(N · S) is the initial permeability, *M* = 23 is an arbitrary material parameter, *ε*_v_ is volumetric strain, and *ψ*(*y*, *h*) is a function dependent on depth.

(4) $$\begin{array}{c} \psi \left(y,h\right)=1+4.3\left(\frac{y}{h}\right)-7.8{\left(\frac{y}{h}\right)}^{2}+3.1{\left(\frac{y}{h}\right)}^{3}. \end{array}$$

#### 2.1.2. Boundary Condition of Articular Cartilage Model

The contact face between the distal end of the femur and the proximal tibia is curved, so contact stress was calculated using Hertz contact theory [25]. Applying hertz pressure to simulate physiological load results in the following expression:

(5) $$\begin{array}{c} F\left(x\right)=\frac{2W}{\pi b}\sqrt{1-\frac{x^{2}}{b^{2}}}\;\left(-b<x<b\right), \end{array}$$

where $b=\sqrt{8FR/\left(\pi E\right)}$ is half of the contact width, *R* = 0.1 m is the equivalent femoral condylar radius, *F* is cyclic loading, and *E* = 10 MPa is equivalent to the elastic modulus. Subchondral bone permeability is very small and approximates to 0, setting the displacement boundary condition:

(6) $$\begin{array}{c} u=v=0,\frac{\partial p}{\partial y}=0. \end{array}$$

The left and right boundaries are symmetrical, the displacement is unconstrained, the pressure is 0, and liquid can flow freely:

(7) $$\begin{array}{c} p=0. \end{array}$$

**Figure 1:**
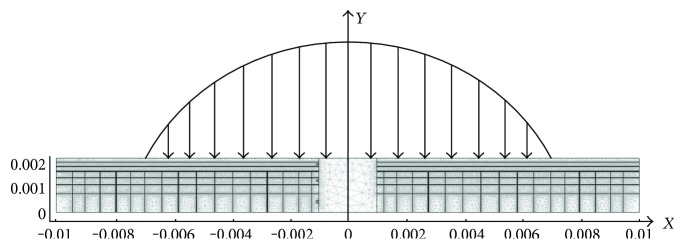
Cartilage defect model and boundary load distribution.

A symmetrical geometric model of cartilage with a length of 2 cm and thickness of 2 mm was established, with the middle area being a defect repair area with a width of 2 mm (Figure 1). The lower boundary was fixed, and the bilateral boundary and the upper surface were allowed to flow freely. A downward cyclic loading was exerted on the upper surface; *W* = *F*_0_sin*ω*t, where *ω* = 2*πf* is the angular frequency, *f* is frequency, and *F*_0_ is the load amplitude. Fibers were horizontal in the superficial layer, transverse and vertical fibers crossing oblique fibers in the middle layer, and with deep layer fibers perpendicular to the cartilage surface.

### 2.2. Finite Element Model of Chondrocytes

**Figure 2:**
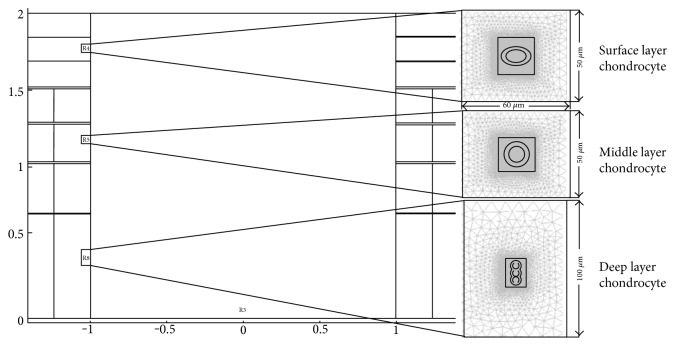
Schematic diagram of chondrocytes in each layer.

The mechanical environment of chondrocytes depends on the macroscopic deformation of cartilage and the location of chondrocytes in cartilage. The chondrocyte model was established according to the cell morphology observed in the staining experiments. The chondrocytes in the superficial and middle layers were oblate and round, with three chondrocytes in a cartilage lacuna forming a cluster (Figure 2). A 100 *μ*m^2^ region around each chondrocyte was defined as a coherence buffer area. The marginal region was extracellular matrix (ECM); the inward transition region was pericellular matrix (PCM) with the chondrocytes in the central area.

#### 2.2.1. Chondrocytes Structural Parameters

The material parameters of the chondrocyte model were taken from the literature [26, 27]: *v*_ECM,1,2,3_ = *v*_PCM,1,2,3_ = 0.2, *v*_CELL_ = 0.4. The remaining parameters are shown in Table 1. In meshing process, the dense of the chondrocyte meshes and the sparse peripheral region of the chondrocyte meshes, the method improves the efficiency of calculation, while ensuring its accuracy.

#### 2.2.2. Validation of Chondrocyte Model

**Figure 3:**
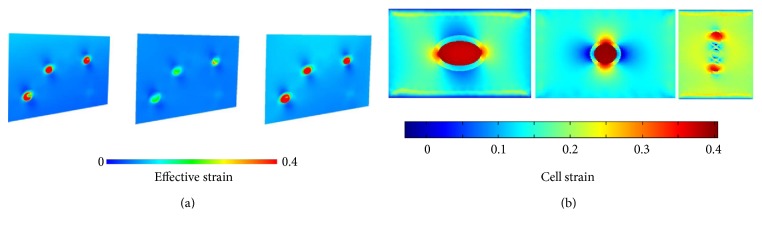
Comparison of simulation results of finite element model of chondrocytes with the literature [18]: (a) results from the literature [18]; (b) results from this paper.

Previous results of the strain analysis of chondrocytes [18] were compared with the simulation results in this paper (Figures 3(a) and 3(b)). It can be seen that strain gradually increased from the ECM to PCM and into the chondrocytes interior, with PCM having the function of strain conduction amplification. The maximum effective strain was 0.4 (Figure 3(a)), strain extremum 0.41 (Figure 3(b)), and the variance ratio of the two values being 2.5%. The strain nephograms were also similar, demonstrating the accuracy of the chondrocyte model.

## 3. Results

### 3.1. Study of the Mechanical Environment of Chondrocytes in the Superficial Layer

In this paper, the artificial cartilage with elastic modulus of 0.1 MPa was used to repair the cartilage defect. Cyclic compressive loading at different frequencies was used to simulate physiological loads, to study the mechanical environment of the chondrocytes, and to explore the mechanical properties of chondrocytes for the repair of cartilage defects.

#### 3.1.1. Variation of Stress-Time Curve at Different Points in the Intracellular Center and PCM

**Figure 4:**
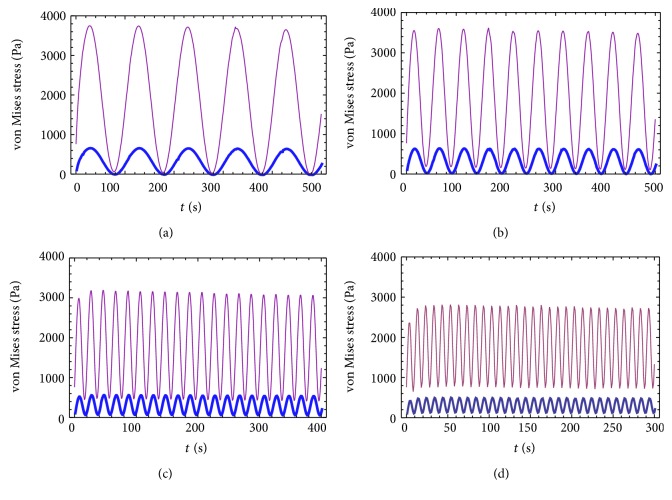
Stress curves at position 1 (a heavy line) and position 2 (a fine line) under cyclic loading at different frequencies: (a) 0.01 Hz; (b) 0.02 Hz; (c) 0.05 Hz; (d) 0.1 Hz.

A uniform stimulation of aggrecan synthesis was observed at frequency of 0.01 Hz, while, at a higher frequency of 0.1 Hz, stimulation was only seen at peripheral radial positions of cylindrical explants [10]. So, this study selected the center of a chondrocyte, labelled position 1, and the PCM directly above position 1, labelled position 2, that were subjected to loading frequencies of 0.01 Hz, 0.02 Hz, 0.05 Hz, and 0.1 Hz. Variations in the stress-time curves at positions 1 (a heavy line) and 2 (a fine line) were obtained within the superficial layer (Figure 4). Maximum stress was observed to be approximately 500 Pa~680 Pa at point 1 and a value 5~6 times greater at position 2 (2800 Pa~3600 Pa) at different frequencies, decreasing gradually over time, indicating that PCM plays a protective and transitional role [16]. At a compressive load frequency of 0.1 Hz, the stress at point 2 was reduced by nearly 1000 Pa compared to the stress at 0.01 Hz. At a low frequency, the response to load at positions 1 and 2 was almost synchronous, but when the frequency was increased to 0.05 Hz, the minimum stress values at positions 1 and 2 were clearly separated. The difference of minimum stress at 0.05 Hz was about 400 Pa and approximately 600 Pa at 0.1 Hz.

The maximum stress decreased at both position 1 and position 2 with increasing frequency, the stress reducing by nearly 170 Pa and 930 Pa at 0.1 Hz compared to that at 0.01 Hz, respectively. However, the minimum stress increased at position 1 and position 2 with increasing frequency, the stress increasing by nearly 160 Pa and 700 Pa at 0.1 Hz compared to that at 0.01 Hz, respectively. At low frequency, positions 1 and 2 respond almost simultaneously to loading. At a frequency of 0.05 Hz, the lowest stress values were separated between positions 1 and 2. The results above indicate that the PCM is sensitive to changes in frequency.

**Figure 5:**
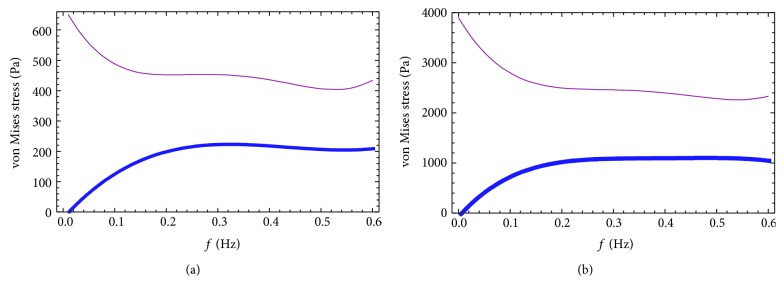
The variation curves of minimum value (a heavy line) and maximum value (a fine line) of stress at different cyclic loading frequencies: (a) position 1; (b) position 2.

The normal walking gait frequency of human is about 0.5–0.7 Hz. Through the analysis of the maximum and minimum values of the stress response curves at position 1 and position 2, which is higher than the frequency of 0.1 Hz, the variation curves of the extreme value stress at different frequencies were obtained (Figure 5). It can be seen that, with the increase of the load frequency, the minimum value of stress at positions 1 and 2 increased, while the maximum value of stress decreased. When the cyclic loading frequency is higher than 0.2 Hz, the extreme value of stress basically keep constant.

#### 3.1.2. The Contours of Stress Distribution of Chondrocytes and PCM

**Figure 6:**
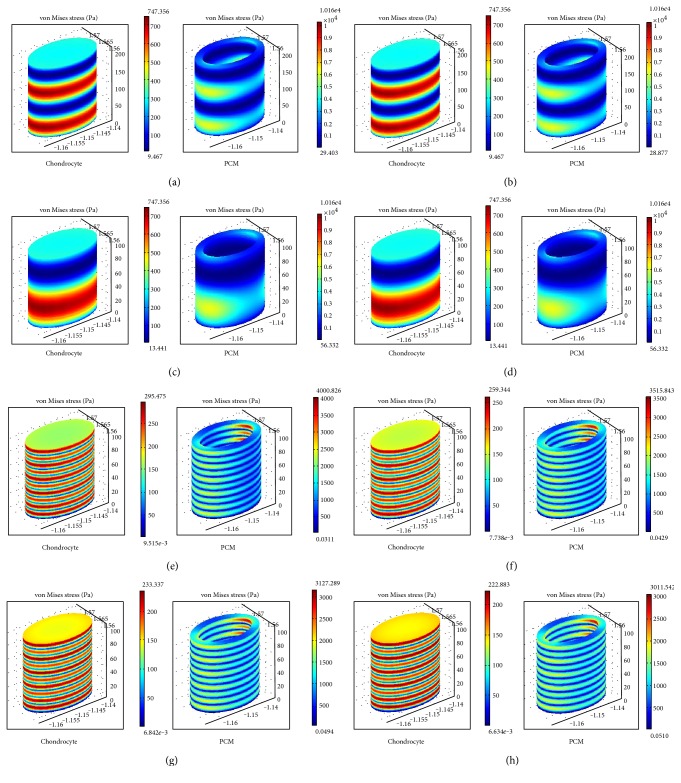
Change in stress in chondrocytes and PCM at different loading frequencies (a)–(d) elastic modulus of artificial cartilage repair: 0.1 MPa, 0.3 MPa, 0.6 MPa, and 0.9 MPa, respectively, at a 0.01 Hz loading frequency; (e)–(h) elastic modulus of artificial cartilage repair: 0.1 MPa, 0.3 MPa, 0.6 MPa, and 0.9 MPa, respectively, at a 0.05 Hz loading frequency.

Following loading for 100 s, the contours of stress distribution in chondrocytes and PCM under cyclic loading were measured at different frequencies (Figure 6). The numerical simulation shows that the stress of cells with different elastic moduli is not obviously different under the same frequency load. Only when the loading frequency is 0.05 Hz, at greater elastic moduli, the stress on chondrocytes is reduced. When artificial cartilage with an elastic modulus of 0.9 MPa was used to repair the cartilage, the maximum stress value in chondrocytes and PCM was approximately 75% than that of the low modulus (0.1 MPa) repair. When applying low frequency (0.01 Hz) loads, the cumulative effect of the load can be fully reflected due to the viscoelastic properties of cartilage. The maximum value of intracellular stress at low frequency loads (0.01 Hz) was higher than that of the high frequency loading (0.05 Hz), the former being 2.53 times the latter. The cumulative effect caused chondrocytes stress to increase with time, but it did not increase indefinitely, and so was beneficial for the protection of chondrocytes from high stress damage and changes in gene expression. Under low frequency loads, using artificial cartilage with larger elastic moduli (0.6 or 0.9 MPa), the stress decreased gradually with increasing load cycle.

#### 3.1.3. Change in Fluid Velocity in Chondrocytes

**Figure 7:**
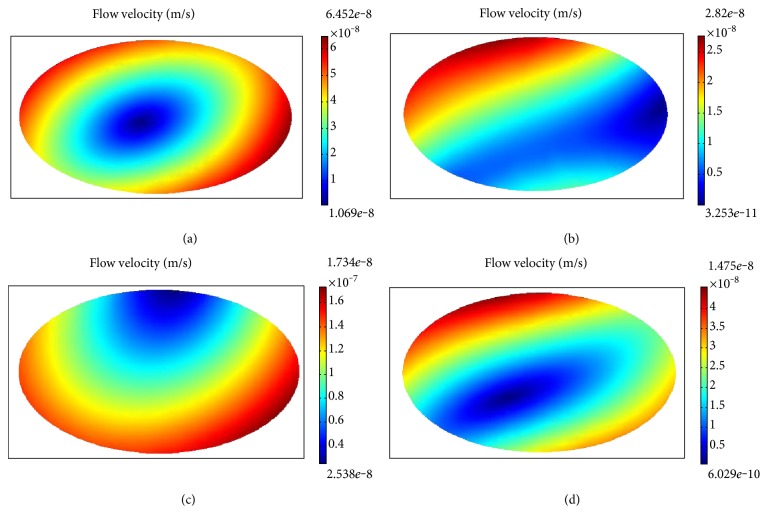
Change in flow velocity at different times under cyclic loading (a) at 4 s, (b) at 9 s, (c) at 14 s, and (d) at 19 s.

Variation in intracellular liquid velocity was most significant in the superficial layer when the loading frequency was 0.1 Hz, as indicated by the distribution maps of intracellular fluid velocity at different loading times (4 s, 9 s, 14 s, and 19 s) (Figure 7). The results show that there were significant differences in the distribution of liquid velocity. In general, the flow velocity in the peripheral area of the chondrocyte was significantly greater than that in the core region. In addition, the simulation also showed that when the cyclic load frequency was lower than 0.05 Hz, the distribution of fluid pressure and velocity in the chondrocytes remained the same but the marginal area was large and the central area was small.

### 3.2. Intracellular Fluid Pressure Distribution at Each Layer

**Figure 8:**
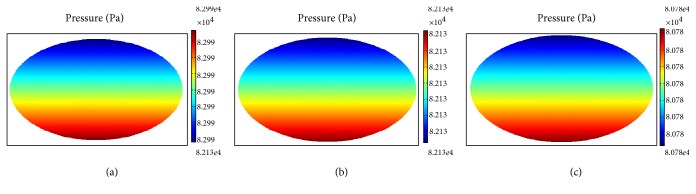
Intracellular fluid pressure distribution at different times in the superficial layer (a) at 15 s, (b) at 16 s, and (c) at 17 s.

**Figure 9:**
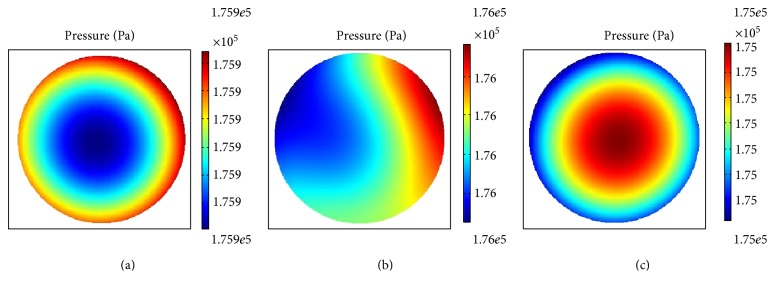
Intracellular fluid pressure distribution at different times in the middle layer (a) at 15 s, (b) at 16 s, and (c) at 17 s.

**Figure 10:**
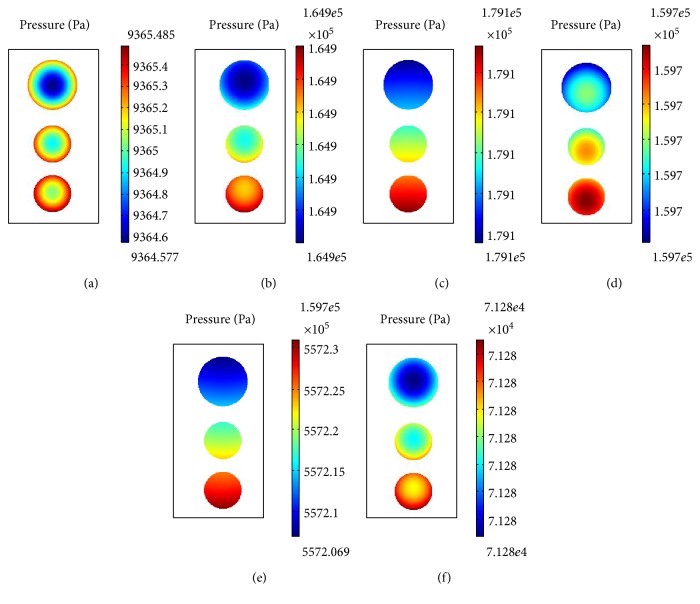
Intracellular fluid pressure distribution at different times in the deep layer (a) at 0 s, (b) at 20 s, (c) at 30 s, (d) at 50 s, (e) at 80 s, and (f) at 100 s.

The distribution of fluid pressure in the superficial, middle, and deep layers under cyclic compression loads at a frequency of 0.02 Hz is shown in Figures 8, 9, and 10, respectively.

Intracellular liquid pressure distribution polarization in the superficial layer resulted in smaller values within the top half and gradually increased in the lower part of the chondrocyte, although the change was not clear (Figure 8). Comparatively speaking, pressure distribution variations were very large in the middle layer. During the process of stress loading over a period of 15–17 s, the compressive stress was concentrated in the periphery of the cell at 15 s, stress concentration transitioning to the cell interior at 17 s with a transition period of liquid pressure distribution at 16 s (Figure 9). The abnormal distribution of cellular fluid pressure may result in gene expression and metabolic abnormalities.

In the deep layer, the pressure within the chondrocytes within the same cartilage lacuna was different (Figure 10). Generally speaking, the largest pressure change was observed in the uppermost chondrocytes and greatest absolute pressure in the lowermost chondrocytes. In the initial stage of loading, intracellular pressure was smaller than the peripheral pressure, but that pressure increased as loading time increased, meanwhile peripheral pressure decreased gradually. The pressure of the liquid changed periodically with loading time, being subject to a certain degree of hysteresis. Conversely, the distribution of pressure varied from internal to the periphery in the lowermost chondrocytes, basically corresponding to the load frequency. Therefore, chondrocytes at different positions within the same cartilage lacuna exhibited different responses to loading.

## 4. Discussion

As it is not possible to receive the results of clinical use of the experimental technology of cartilage repair over a short time, it is very important to ascertain the mechanical environment of chondrocytes and their surroundings in vivo using multiscale finite element numerical simulation analysis [16]. In this paper, the micromechanical environment of the chondrocytes in a defect repair zone under cyclic compressive loading was studied for the first time. The simulation results can help explore the causes of tissue-engineered cartilage failure and guide clinical rehabilitation.

Using a multiscale method, the biomechanical properties of chondrocytes in intact articular cartilage and tissue from medial meniscectomy were studied under joint load [16–18]. The focus of study in this paper was on the response of chondrocytes to compressive loading (static or cyclic). In addition, the difference in shape and distribution of chondrocytes in cartilage lacunae in different layers was considered using modeling. According to our simulation, the mechanical environment of chondrocytes is depth-dependent under cyclic loading and the properties of the defect repair significantly alter the mechanical environment of the repair zone, factors not previously considered in former studies.

The mechanical response of chondrocytes depends on the applied load characteristics. According to our findings, the effect of cyclic loading on the micromechanical environment of chondrocytes was different in the different layers in the defect repair zone. The current simulations may be used as a guideline to predict the effect of cyclic variation on the micromechanical environment of chondrocytes as a function of position.

The results of studies of the biomechanical environment of chondrocytes have not been reported during previous repairs of defects using tissue engineering. Thus, the contours of strain distribution were compared with those obtained from high-throughput multiscale 3D models of chondrocytes, with at most differences of 2.5% [18], to ensure validity of the results of subsequent analysis.

In this paper, there are still some deficiencies, ignoring the fine structure of the cell and regarding chondrocytes as a continuum. In fact, the cell structure can be regarded as a type of tension integration model comprising an inner liquid core and organelles [14]. The liquid phase accounts for 95% of the cell, the solid phase 5%. A chondrocyte stent is composed of a fibrous structure of protein filaments and microtubules, similar to an umbrella frame supporting the umbrella surface. Our assumption was that the artificial cartilage was fully bonded with the host cartilage, whereas cracking and incomplete adhesion may characterize the actual situation. The selected load frequency range is smaller than that experienced in vivo. Subsequent studies need also to consider the effects of different shapes and depth of repair on the biomechanical environment of chondrocytes.

## 5. Conclusions

In this paper, the changes in stress and fluid pressure in chondrocytes within different regions of a repaired area of cartilage were analyzed. The mechanical environment of each layer of chondrocytes in the host cartilage was inevitably affected. The two-phase solid and liquid structure of articular cartilage provides it with viscoelastic properties. Due to the cumulative effect of the cartilage structure, the lowest frequency (0.01 Hz) of the cyclic load caused increased notable stress concentration in chondrocytes in the superficial layer, while the amplitude of cyclic stress response curve of chondrocytes reduced with increasing frequency. There was no significant effect of cyclic loading on the distribution of intracellular fluid pressure in the superficial layer, but the influence of cyclic loading on the distribution of liquid pressure in the middle and deep layers was significant. However, the change in the mechanical environment of chondrocytes in each layer is likely to result in damage to the chondrocytes or to result in abnormal gene expression.

In general, the elastic modulus of artificial cartilage had little effect on the mechanical environment under the same cyclic loading frequency. Clinically, following the repair using tissue-engineered cartilage, the appropriate loading frequency can be used to produce a reasonable mechanical environment for the chondrocytes, which can help patients to take appropriate exercise for rehabilitation.

## Figures and Tables

**Table 1:** Mechanical parameters of chondrocytes.

Position	*E*_ECM_ (MPa)	*E*_PCM_ (KPa)	*E*_CELL_ (KPa)	*k*_CEM_/10^14^ (Ns·m^−4^)	*K*_PEM_/10^15^ (Ns·m^−4^)	*K*_CELL_/10^15^ (Ns·m^−4^)
Superficial layer	0.4	40	4	2	1	2
Middle layer	0.3	30	3	2	1	2
Deep layer	0.2	20	2	2	1	2
